# Pregnenolone Sulfate: From Steroid Metabolite to TRP Channel Ligand

**DOI:** 10.3390/molecules181012012

**Published:** 2013-09-27

**Authors:** Christian Harteneck

**Affiliations:** 1 Department of Pharmacology and Experimental Therapy, Institute of Experimental and Clinical Pharmacology and Toxicology, Eberhard Karls University, Tübingen 72074, Germany; 2 Hospitals and Clinics, Interfaculty Center of Pharmacogenomics and Drug Research, University of Tübingen, Tübingen 72074, Germany; E-Mail: christian.harteneck@uni-tuebingen.de; Tel.: +49-7071-2972-271; Fax: +49-7071-29-4942

**Keywords:** neurosteroids, pregnenolone sulfate, steroid metabolism, ion channel modulation, TRP channels

## Abstract

Pregnenolone sulfate is a steroid metabolite with a plethora of actions and functions. As a neurosteroid, pregnenolone sulfate modulates a variety of ion channels, transporters, and enzymes. Interestingly, as a sulfated steroid, pregnenolone sulfate is not the final- or waste-product of pregnenolone being sulfated via a phase II metabolism reaction and renally excreted, as one would presume from the pharmacology textbook knowledge. Pregnenolone sulfate is also the source and thereby the starting point for subsequent steroid synthesis pathways. Most recently, pregnenolone sulfate has been functionally “upgraded” from modulator of ion channels to an activating ion channel ligand. This review will focus on molecular aspects of the neurosteroid, pregnenolone sulfate, its metabolism, concentrations in serum and tissues and last not least will summarize the functional data.

## 1. Introduction

**Figure 1:**
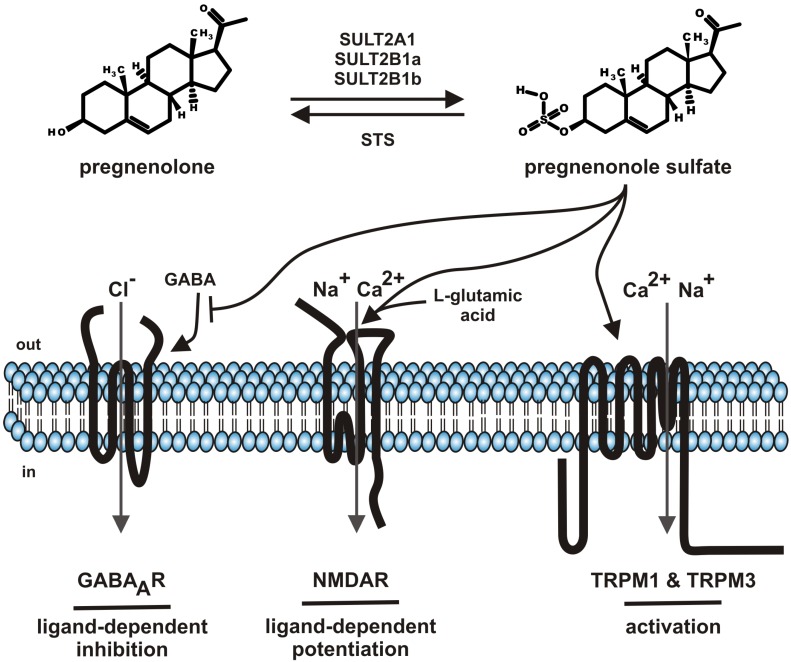
Summary of pregnenolone sulfate synthesis, metabolism and functions.

SULT: cytosolic sulfotransferase enzymes; STS: steroid sulfatase; GABA: gamma-aminobutyric acid; GABA_A_R: ionotropic receptor of gamma-aminobutyric acid; NMDAR: ionotropic receptor of l-glutamic acid, N-methyl-D-aspartate subtype; TRPM: members of the melastatin-like transient receptor potential channel (TRPM) subfamily; out: outside the cell, extracellular; in: inside the cell, intracellular.

Steroids comprise a group of chemical related hormones mediating their function by modulation of transcriptional activity. Steroids like cortisol, estradiol, testosterone, or aldosterone bind to intracellular cytosolic glucocorticoid receptor, estrogen receptor, androgen receptor or mineralocorticoid receptor, respectively. The receptor-hormone complex subsequently translocates from the cytosol to the cell nucleus and regulates transcriptional activity. The genomic effects of steroid hormone application are detectable with some delay. Beside the genomic effects of steroids, fast, membrane-delimited non-genomic effects of steroids have been described [1]. The question of specificity dominated this field. On one hand, reports showed that such effects, e.g., modulation of GABA_A_ receptor by neuroactive steroids depends on the cholesterol content of the plasma membrane by using methyl-β-cyclodextrin as tool to interfere with the endogenous cholesterol content of the plasma membrane [2]. On the other hand, a growing spectrum of steroid compounds, intermediates from steroid synthesis, metabolites (sulfate-, acetate conjugates), most of them designated as neurosteroids, helped to unravel the diversity of specific effects of membrane-delimited steroid actions [3,4,5]. This review will focus on one particular neurosteroid, pregnenolone sulfate (PregS, Figure 1) as it has been functionally “upgraded” from modulator of ion channels to an activating ion channel ligand by the discovery that PregS activates TRPM3, an ion channel of the TRP superfamily [6]. The review will sum data on metabolism, serum and tissue concentrations and functional data on PregS. 

## 2. Pregnenolone Sulfate—Synthesis and Metabolism

Steroid sulfates are formed by sulfonation of steroids by cytosolic sulfotransferase enzymes (SULT) [7,8]. So far more than 40 distinct cytosolic sulfotransferase enzymes have been identified from which three enzymes (SULT1E, SULT2B1, SULT2A1) selectively sulfonate steroids with distinct profiles of steroid specificities. SULT1E, SULT2B1, SULT2A1 mediate the sulfonation of estrogen, nonaromatic steroids and sterols, respectively. PregS is generated by SULT2A1, SULT2B1a and SULT2B1b (Figure 1) [8]. Based on the NCBI EST profiles, SULT2A1 mRNA is transcribed preferentially in the adrenal gland, liver, parathyroid gland, whereas the SULT2B1 transcripts show reduced mRNA counts widely distributed in many tissues like ascites, brain, connective tissue, eye, heart, intestine, kidney, lung, mammary gland, pancreas, placenta, prostate, and testis. With respect to distribution of sulfotransferases in brain, recently the frog SULT enzyme has been localized to the GnRH-innervated regions of the magnocellular nucleus and anterior preoptic area [9]. The broad tissue distribution of SULT enzyme argues for the generation of PregS in many tissues and its different tissue–specific roles.

Differences in SULT2B1 enzymes result from splice events leading to two different mRNAs encoding N-terminally diverging proteins SULT2B1a and SULT2B1b [10]. The splice differences are accompanied by distinct substrate selectivities. SULT2B1b is highly selective for cholesterol being the cholesterol sulfate forming enzyme in the skin, liver, and elsewhere, SULT2B1a ignores cholesterol and prefers pregnenolone [8]. Pregnenolone is synthesized from cholesterol by a mitochondrially localized cytochrome P450 side-chain cleavage enzyme (scc, CYP11A1) [4,11]. This central step in steroidogenesis is rate limiting and essential. Diethylstilbestrol, tamoxifen, cocaine, and other drugs have been described to interfere with steroidogenesis by downregulation of cytochrome P450 side-chain cleavage enzyme [12,13,14]. SULT2B1a expression is regulated by l-glutamic acid via AMPA receptor-mediated NO signaling [15], which is interesting as PregS potentiates l-glutamic acid transduction via N-methyl-d-aspartate receptors (NMDAR) (see below). 

In the context of xenobiotic metabolisms, sulfonation is discussed as an endpoint leading to increased water solubility and enhanced renal clearance of the metabolite [16]. However, steroid sulfates additionally represent ion channel modulators and storage intermediates. As source and building block for steroid synthesis, steroid sulfates had to enter cells for sulfohydrolase reaction. The passive passage and diffusion of steroid sulfates are hampered by hydrophilicity of the sulfate moiety, for which reason cellular uptake of steroid sulfates is dependent on transporters (see below) [17,18]. The steroid releasing reaction is mediated by steroid sulfatase (STS) an enzyme encoded by a X-chromosomally localized gene [19,20,21]. The physiological impact of STS can be anticipated by the fact that the congenital deficit of STS is causing X-linked recessive ichthyosis [22]. The disease affecting males in an incidence of 1: 2000–6000, is characterized by dry, scaly skin due to deletions or mutations in the STS gene and subsequent deficits in steroid hormone function [22]. In addition, biochemical studies using radioactive labeling approaches showed the release of pregnenolone and subsequent steroid metabolites from PregS [23,24,25,26].

## 3. Pregnenolone Sulfate Levels in Serum and Tissues

Human serum PregS concentrations change during lifespan from µM concentrations during birth to nM concentrations in adults [27]. The mean PregS concentrations during birth are 3.06 µM (♀) and 2.5 µM (♂) in the umbilical cord and 1.85 µM (♀) and 2.2 µM (♂) in fetal serum [27]. The PregS levels decline rapidly during the first two weeks to 1.02 µM (♀) and 1.4 µM (♂), reaching levels of 56 nM (♀) and 32 nM (♂) in the first year [27]. These levels are more or less stable during childhood and then double during adolescence, reaching levels of 130 nM (♀) and 140 nM (♂) in adults [27]. The high PregS levels during birth are indicative for the role of PregS during pregnancy and birth, for which reason the PregS levels during this period induced a variety of studies analyzing PregS levels during pregnancy and birth [28,29,30,31,32,33,34,35]. PregS in maternal plasma (P), umbilical artery (UA), umbilical vein (UV) rises from 930 nM (P), 3.8 µM (UA), 3.6 µM (UV) during pregnancy to 1250 nM (P), 4.8 µM (UA), 3.9 µM (UV) during birth [29,33]. A study in twins revealed that PregS plays an important role for the onset of parturition [34]. The increase of PregS before and during parturition is so far published a unique feature in humans. The increase in concentration prior parturition seems to be a species–specific regulation mechanism, as in contrast in sheep, the PregS concentrations rise during pregnancy and rapidly decline from day 3 before birth in fetal as well as maternal plasma [36]. However, the absence of large amplitudes during the oestrous cycle in seems to be a common feature in mammals. PregS levels in ewes show only small deviations from the mean values [37]. Values for women are only available from women suffering from premenstrual syndrome also show only small deviations in PregS levels during the oestrous cycle [38]. In contrast to the data situation on PregS in women during the oestrous cycle, several studies analyzed PregS occurrence in testis from men who have undergone orchiectomy due to prostatic carcinoma [39,40,41]. Whereas the peripheral PregS serum levels are in the range of the concentrations mentioned above, the PregS concentrations in spermatic venous blood were 3–4 fold increased [41]. In testis tissue, PregS concentrations reach 2.8 to 4.0 µmol/kg, in vas deferens and epididymis and 1.4 µmol/kg and 0.76 µmol/kg, respectively [39,40]. 

The dynamic regulation of PregS levels during pregnancy illustrates that steroid as well as neurosteroid levels can change due to physiological adaptation. Adrenal stimulation in children increased PregS parallel to cortisol levels enabling the usage of PregS in diagnosis of various adrenal and pituitary diseases in children [27,42]. A more general role of PregS became evident by reports of changed PregS levels in disease states [43,44,45,46,47]. For example, PregS serum concentrations are reduced in hypothyroidism and 7fold increase in hyperthyroidism [46]. In patients suffering from rheumatoid arthritis, PregS levels are 4fold reduced [45]. 

The occurrence of PregS in the brain has been a matter of debate influenced by imponderabilities of the analytic methods summarized in a review by Schumacher and coauthors [48]. The impact of PregS as neurosteroid in disease has been documented by a report showing that PregS tissue concentrations decreased in Alzheimer patients [49]. The correlation of high levels of key proteins implicated in the formation of plaques and neurofibrillary tangles with the decreased levels of PregS and other neurosteroids suggest a possible neuroprotective role of PregS in Alzheimer disease [49]. In human brain, PregS concentrations vary from 5 to 42 nmol/kg PregS, depending on brain region, with the highest concentrations of 35 and 42 nmol/kg PregS in striatum and hypothalamus, respectively [49]. Comparable values were published for rat brain PregS concentrations of 45 nmol/kg [50,51]. Interestingly, a study analyzing neurosteroid levels in developing rat brain upon fetal alcohol exposure revealed that fetal alcohol exposure by maternal ethanol consumption increase PregS level in brain during fetal development and last till postnatal day 5 [52]. Enhanced PregS levels were restricted to the fetal brain. The maternal brain, maternal blood, placenta, and fetal liver were not affected excluding secondary accumulation of peripherally-produced steroids [52]. In the context with recent finding of PregS-induced, TRPM3-mediated potentiation of synapse formation in Purkinje cells (see below), fetal alcohol exposure leading to enhanced PregS levels might be responsible for an inadequate neuronal architecture. Daily subcutaneous application of 20 mg/kg PregS protected the survival and maturation of newborn neuronal cells in APP/PS1 transgenic mice, a mouse model for Alzheimer disease [53]. Concurrently, the spatial cognitive performance was improved in the PregS-treated mice [53].

## 4. Transporter—Cellular Uptake of Pregnenolone Sulfate

It is unlikely that pregnenolone sulfate is capable of easily crossing the plasma membrane due to the hydrophilic sulfate moiety. On the other hand, for its function as source for pregnenolone and subsequent synthesis of steroid hormones like estradiol, progesterone *etc.*, it is indispensible that the substrate (PregS) enters the cytosol to come into contact with the cytosolic localized sulfohydrolases. The transmembrane transport can be facilitated by a variety of transporter proteins [17,18]. The organic anion-transporting polypeptide (OATP-B) has been cloned from placenta as steroid conjugate transporting protein [54]. OATP-B is nowadays classified as solute carrier organic anion transporter (SLCO2B1). OATP-B/SLCO2B1 is expressed in many organs like liver, mammary gland, brain and intestine, whereas other organic anion transporting polypeptides (OATP)-A or C, are limited to special tissues (such as liver or brain) [55]. OATP-B/SLCO2B1 has been shown to be localized in the placental basolateral membrane compartment mediating estrone-sulfate uptake being inhibited by PregS [54]. The transport properties are quite complex as later studies showed that OATP-B/SLCO2B1 mediates PregS uptake, which is further enhanced in the presence of progesterone, whereas PregS blocked 28% ± 7% and 43% ± 7% of the estron sulfate uptake and dehydroepiandrosterone sulfate uptake, respectively [55]. 

A high capability to transport PregS has also been shown for a transport protein of another subfamily, the sodium-dependent organic anion transport (SOAT) nowadays, solute carrier family 10 (sodium/bile acid cotransporter) (SLC10A6) [56,57]. With respect to function of PregS as neurosteroid, the organic solute transporter OSTα-OSTβ is of particular interest [58]. The transport protein of the organic solute transporter family (SLC51) is expressed in the human tissues liver, ovary, adrenal gland and hippocampus. By laser capture microdissection RT-PCR analysis, mRNA transcription of OSTα-OSTβ was detected in Purkinje cells and cells in the CA region of the hippocampus murine Purkinje and hippocampal cells, the steroidogenic cells of the brain [58]. PregS as well as DHEAS represent relatively high affinity substrates for OSTα-OSTβ/SLC51. Estron sulfate uptake in the presence of PregS or DHEAS, results in inhibition of half of the transport [58]. 

## 5. Molecular Targets of Pregnenolone Sulfate

A broad range of ion channels have been shown to be modulated by PregS. Many reports remained anecdotally like the modulation of potassium channels [59,60] or nicotinic acetylcholine receptor [61] or voltage-gated sodium channels [62]. In contrast (Figure 1), the negative modulation of GABA_A_ chloride channels, the positive modulation of glutamate response by NMDA receptors and the activation of TRPM1 as well as TRPM3 channels are well established and will be the topics of the following chapters (Table 1).

**Table 1:** Molecular targets of pregnenolone sulfate.

Molecular target	Mode of action	EC_50_/IC_50_ values	Physiological impact
GABA_A_ channel	inhibition	IC_50_ 7.2 µM	long term potentiation, memory & learning, anxiolysis, general anesthesia epilepsy, musclar cramps
		(GABA 3 µM) [63]	
NMDA receptor	potentiation	EC_50_ 33 µM	neuronal development, synapse formation
		(NMDA 5 µM) [64]	
TRPM1	activation		melanocyte function, melanin synthesis, phototransduction
TRPM3	activation	EC_50_ 23 µM (– 80 mV) [6]	pain modulation, insulin secretion, neuronal development
		EC_50_ 12 µM (+ 80 mV) [6]	
		[Pregnenolone EC_50_ 15 µM	
		(–80 mV)] [6]	
		[Pregnenolone EC_50_ 14 µM	
		(+80 mV)] [6]	

### 5.1. GABA_A_ Channels

The clearance of barbiturate-induced hypnosis by PregS was the starting point for the characterization of interaction of PregS with the GABA_A_ receptor [65]. Subsequent studies showed binding to and functional inhibition of GABA_A_ receptors [66,67]. The modulation of GABA_A_ receptors by PregS seems to be limited to a distinct subunit composition [68]. GABA_A_ receptors reassembled from α_1,2,3_ + β_1_ + γ_2_ are sensitive to PregS-mediated inhibition [68]. The effect of PregS on GABA_A_ inhibition is independent of ethanol, whereas modulation by allopregnanolone, another neurosteroid, was affected [69]. The neurosteroid effect on GABA_A_ receptors is phylogenetically conserved. UNC-49, the *C. elegans* orthologue of mammalian GABA_A_ receptors, is modulated by PregS [70]. The transcription of two splice variants of *C. elegans* UNC-49 resulting in the translation of UNC-49B and UNC-49C differing in neurosteroid sensitivity allowed to map the region of neurosteroid interaction [70]. Site-directed mutagenesis und functional analysis showed that a region of 6 residues in the first transmembrane domain (M1) mediates PregS modulation [70]. However transferring these finding to the mammalian GABA_A_ receptor orthologue showed quite different results [71]. Therefore, it is unclear whether the PregS interaction site is localized elsewhere or formed by M1 in conjunction with structure-stabilizing amino acids of the other transmembrane segments (M2, M3, M4). GABA as neurotransmitter plays a central role in the neuronal network allowing the pharmacological modulation to achieve anxiolysis and general anesthesia or interfere in epilepsy or muscular cramps. Therefore, a variety of central as well as peripheral effects of PregS have been linked to its ability to interfere with GABA function [5].

### 5.2. NMDA Receptors

PregS is an allosteric modulator at the l-glutamic acid receptor [63]. With respect to selectivity, it is remarkable that selectively N-methyl-d-aspartate (NMDA) receptors are modulated in a positive way, whereas α-amino-3-hydroxy-5-methyl-4-isoxazolepropionic acid receptors (AMPAR) or kainate receptors are negative regulated [63]. The half-maximal concentration of PregS (EC_50_ value) in the presence of 5 µM NMDA is 33 µM (Table 1) [64]. The effects of PregS can be shifted to nanomolar concentrations in the presence of the NMDA receptor antagonists, ifenprodil or dizocilpine (MK-801) [72,73]. NMDA receptors assemble as obligate heteromers that may be drawn from NR1, NR2A, NR2B, NR2C, NR2D, NR3A and NR3B subunits [74]. Splicing events increase variability of the NMDA receptor family [74]. The magnitude of PregS-mediated effect tested on the NR1a/NR2A and NR1a/NR2B heteromers vary showing a larger effect on NR1a/NR2A [75]. Further studies revealed that PregS potentiated NMDA-induced currents of NR1/NR2A and NR1/NR2B receptors, whereas it was inhibitory at NR1/NR2C and NR1/NR2D receptors [76,77]. The effect of PregS on NMDA receptors is dependent on protein phosphorylation as protein kinase inhibitors as well as protein phosphatase inhibitors can interfere with the PregS-mediated modulation of NMDA receptors [78]. Additionally, NMDA receptor function is modulated by several endogenous molecules, including zinc, polyamines, protons, and sulfated neurosteroids. Zinc, polyamines, and phenylethanolamines exert their modulatory role by inferring with inhibition via protons. Proton-dependent inhibition depends on aspartate residue (D690 in NR1B) in the extracellular loop adjacent to the M3 transmembrane domain [79]. Detailed analysis showed corresponding domains in NR2A and revealed that additional residues are involved in proton-dependent inhibition [80]. PregS binding was also mapped to S2 extracellular loop however to another region adjacent to the M4 transmembrane domain. [81], which argues a unique mechanism for NMDA receptor enhancement independent of the proton sensor. In addition to the acutely affecting modulation of NMDA receptor, PregS enhance NMDA receptor function by increasing NMDA receptor insertion in the cell surface [82]. The enhanced integration of NMDA receptor proteins in the plasma membrane is mediated via a non-canonical, pertussis toxin–sensitive, G protein–coupled, and calcium-dependent mechanism independent of NMDA receptor activation [82]. 

Glutamate receptors play a central role in neuronal regulation [83]. Therefore, a variety of reports describe PregS-mediated potentiation of NMDA receptors which are involved in many processes like learning and memory [84,85,86], central regulation of body functions [87] or effects of drugs like ethanol [87], opioids [88]. In many of these reports, the effects have been assigned based on the use of a spectrum of pharmacological tools designated to interfere with NMDA receptors. However, some cautions is appropriate with respect to findings that PregS activates TRPM1 and TRPM3 [6,89]. Starting from the finding that PregS potentiates spontaneous glutamate release onto neonatal Purkinje cells during a period of active glutamatergic synapse formation [90], we showed that the potentiation is mediated by the direct activation of TRPM3 channels expressed in Purkinje cells [91].

### 5.3. TRP Channels (TRPM1, TRPM3)

TRPM1 and TRPM3 are members of the mammalian melastatin-like transient receptor potential (TRPM) channel subfamily including eight members based on their homology to melastatin (TRPM1), a putative tumor suppressor involved in the pathophysiology of melanoma [92]. Proteins of the TRPM channel, the classic transient receptor potential channel (TRPC), the vanilloid-like transient receptor potential channel (TRPV), the ankyrin-rich transient receptor potential channel (TRPA), the transient receptor potential channel of the polycystin group (TRPP), and the transient receptor potential channel of the mucolipin group (TRPML) constitute the superfamily of TRP channels [93]. The ion pore of TRP channels is formed by a homo- or heterotetrameric complex of four TRP channel proteins. The multimerization described so far showed that heteromerization is possible in the limited range of closely related TRP channels [94,95,96]. TRPM proteins assemble into ion conducting channels that respond to a variety of stimuli including temperature, osmolarity, various chemical signals, change in membrane voltage, oxidative stress, and intracellular calcium [97]. Studies on TRP channel function are currently handicapped by the situation that the blockers available mostly represent pan TRP channel blocker hindering direct assignment of TRP channel-mediated functions [98,99]. 

**TRPM3** is a polymodal ion channel activated by a variety of different stimuli like hypotonicity [100], sphingolipids [101], steroids [6,102], nifedipine [6], and heat [103]. The steroid-dependent modulation of TRPM3 is complex. Pregnenolone sulfate, pregnenolone and epipregnanolone sulfate stimulate TRPM3 activity whereas progesterone inhibits PregS-stimulated TRPM2 activity [6,102,104]. The concentration-response curves of PregS and pregnenolone reveal comparable potency of both steroids (Table 1), whereas both activating ligands showed dramatic differences in efficacy. The intrinsic activity of PregS is more than ten-fold higher than the intrinsic activity of pregnenolone [6]. With respect to the hydrophilicity of the sulfate moiety, it is interesting that TRPM3 is activated selectively by extracellular application of PregS, intracellular infusion of PregS via the patch pipette failed to induce TRPM3-mediated currents [6]. The activation of TRPM3 is remarkable as the temperature profile shows a continuous increase in TRPM3 activity in the range of 27 °C to 43 °C [103], which is contrast to TRPV1 showing increased activity beyond a threshold of 40 °C [105]. However, the most fascinating feature of the heat-dependent modulation of TRPM3 activity is the sensitization towards PregS [103]. At normal human body temperature, TRPM3 can be activated by PregS concentrations in the range of 100 to 500 nM corresponding to physiologic PregS serum concentrations (see above) [103]. Electrophysiological characterization revealed permeability of TRPM3 for calcium, sodium, magnesium, manganese and zinc ions [100,101,106,107]. Expression profile and subsequent functional analysis provides evidence for TRPM3 function in vascular smooth muscle contraction [108], modulation of glucose-induced insulin release from pancreatic islets [6,109], detection of noxious heat in dorsal root ganglia [103], and activation of oligodendrocytes and neurons in developing brains [91,110]. 

**TRPM1**, the founding member of the TRPM channel subfamily was discovered in a screen looking for transcriptional regulation in melanoma [92,111]. The absence of TRPM1 in malign melanoma guided the authors to name the TRP channel identified melastatin. Difficulties in cDNA cloning and expression as well as occurrence of many splice variants hampered the characterization of TRPM1 [112]. The protein was mainly studied in the context of melanocytes [113] until its role in mammalian phototransduction was discovered [114]. In electroretinograms (ERG) of TRPM1^−/−^ mice the so-called b-wave, the measure for ON bipolar cells was missing [114]. Immunohistochemistry revealed expression of TRPM1 in rod ON bipolar cells and initiated a series of studies characterizing TRPM1 in the context of glutamate-induced signal transduction comprising metabotropic glutamic acid receptor (mGluR6), heteromeric Go proteins, and RGS proteins [115,116,117,118,119,120]. As TRPM1 like TRPM3 is activated by PregS [89] it is interesting to note the ability of retina for neurosteroidogenesis, which has been shown in rat retina [121]. Furthermore, it is possible that the neurotoxic effects in retina induced by PregS [122,123,124] are mediated by TRPM1-mediated calcium entry. 

## 6. Conclusions

The aspects summarized here illustrated the versatile features of PregS from pregnenolone metabolite to source of pregnenolone as mother of steroids in subsequent steroid synthesis pathways to modulatory impact on ion channels. The latter is still matter of debate in the cases of GABA_A_ channel and NMDA receptor activation. The nanomolar concentrations found in most tissues and situations on one hand and the micromolar concentrations used in the experiments on the other are critically discussed with respect to whether or not the experiments will mimic physiologic relevant pathways. Even the experiments showing effect of PregS in nanomolar concentrations in the presence of NMDA receptor blocker represent not really physiologic conditions. Currently, it could not be excluded that the modulation of GABA_A_ channels and NMDA receptors by PregS show a different profile in experiments performed under physiologic temperatures as it has been shown for TRPM3. At all the unsolved questions in this debate, PregS is an interesting ligand and provide a broad field for future studies dissolving the physiologic role of PregS in the brain and peripheral organs. 
